# Determination of immunogenicity of an inactivated ND-vaccine developed experimentally with Newcastle disease virus (Genotype VII.2) local isolates of Bangladesh

**DOI:** 10.3389/fimmu.2024.1482314

**Published:** 2024-11-06

**Authors:** Mohammad Aynul Haque, Md. Enamul Haque, Mst. Kohinoor Parvin, Md. Mostofa Kamal, Tanbin Rubaiya Islam, Mohammad Sadekuzzaman, Md. Ariful Islam, Mst. Minara Khatun, Muhammad Tofazzal Hossain, Mohammad Asir Uddin, Sham Soun Nahar, A. K. M. Khasruzzaman, Md. Alimul Islam

**Affiliations:** ^1^ Department of Microbiology and Hygiene, Bangladesh Agricultural University, Mymensingh, Bangladesh; ^2^ Central Disease Investigation Laboratory, Department of Livestock Services, Dhaka, Bangladesh; ^3^ Livestock Research Institute, Department of Livestock Services, Dhaka, Bangladesh

**Keywords:** NDV genotype VII.2, inactivated ND vaccine, immunogenicity, protective potentiality, antibody titers

## Abstract

**Background:**

Newcastle disease virus (NDV) genotype VII severely affects poultry, causing respiratory and neurological symptoms with a high rate of morbidity and mortality. The research aimed to develop an inactivated ND vaccine using local isolates (Genotype VII.2) and assess its immunogenicity compared to other commercial live ND vaccines.

**Methods:**

An inactivated vaccine using a candidate NDV (GenBank: OR924274.1) was developed according to WOAH guidelines following inactivation, sterility, purity, and safety tests. The birds were vaccinated through subcutaneous (SC) and intramuscular (IM) routes using three doses (0.25, 0.5, and 1.0 ml/bird). Immunogenicity and protective potentiality of the experimentally developed inactivated ND vaccine and live commercial ND vaccine (intra-ocularly/IO) were compared by challenge studies using three vaccination schedules: killed-followed-killed, live-followed-killed, and live-followed-live.

**Results:**

The birds vaccinated with 1.0 ml/bird SC showed higher antibody titers compared to those of IM-vaccinated groups. Birds vaccinated with the live-followed-killed commercial ND vaccines had slightly higher antibody titers compared to those vaccinated with killed-followed-killed and live-followed-live vaccines. Birds vaccinated with the killed-followed-killed ND vaccine showed a higher protection rate (100%) compared to live-followed-killed (83±5.77%) and live-followed-live (57±5.77%) vaccines. Birds vaccinated with killed-followed-killed group showed a slower decline rate of antibody titers than other groups. This regimen provided significantly better immunity, highlighting its potential in controlling ND outbreaks in Bangladesh's poultry.

**Conclusion:**

The study found that the inactivated ND vaccine, developed with the locally circulating isolate of genotype-VII.2 of NDV, might play an important role in effective control and management of ND in the commercial poultry population in Bangladesh.

## 1 Introduction

Newcastle disease (ND) is a highly infectious viral disease that carries huge impacts on a diverse array of bird species and has substantial economic consequences for the global poultry industry (1). The Newcastle disease virus (NDV) belongs to the genus *Orthoavulavirus* of the family *Paramyxoviridae* (2, 3). There are different strains, or genotypes, of the virus, and one of the most common and virulent strains is genotype VII, especially circulating in Asia and Africa (4). ND poses a significant threat to the poultry sector in Bangladesh, which plays a crucial role in the country’s agricultural economy and supports the livelihoods of millions of people (5).

To control and prevent ND in commercial poultry farmers have to depend mainly on vaccination. In Bangladesh, the management of ND in commercial poultry involves using different types of ND vaccines (6). These vaccinations are specifically formulated to stimulate robust immune responses, providing defense against the highly infectious strains of NDV that are widespread in different areas in a country (7). The main categories of vaccinations employed include live attenuated vaccines, inactivated vaccines, and recombinant vector vaccines (8). NDV strains that have been weakened but can still multiply somewhat are used to produce live attenuated vaccines. Strains of NDV including LaSota, B1, and F strains are frequently utilized for the development of live attenuated ND vaccines (9). Vaccines that have been inactivated are known to elicit strong humoral and cell-mediated immune responses, providing efficient defense. Usually, the live attenuated ND vaccines are applied as eye drops, aerosol, or drinking water, which guarantees quick and simple administration (10). The NDV particles of the inactivated vaccines have been rendered non-infectious by chemical agents like formalin, β-Propiolactone, and Binary ethylenimine (BEI). Injectable inactivated vaccines are frequently adjuvanted to boost the immune response (11). Killed vaccines are frequently serve as booster doses after the initial live vaccination, and they are very helpful in providing long-lasting protection. Live attenuated NDV antigens are delivered via recombinant vector vaccines using viral vectors like herpesvirus or fowlpox. They have the benefit of potent immune responses without having any danger like live attenuated vaccines. They can also be made to target different infections, offering more comprehensive protection (12). The efficacy of a vaccine is heavily influenced by the degree of similarity between the vaccine strain and the strains currently in circulation (13). In recent years, the appearance of several NDV genotypes, particularly genotype VII, has presented difficulties for the current vaccination regimens due to a mismatch of the vaccine and circulating strain of the viruses. As a result, there has been a need to develop vaccinations that are specially designed to target the specific strains of viruses that are now prevalent in different regions (14).

The commercial poultry industry in Bangladesh has been struggling for the last decades with repeated occurrences of ND, which frequently results in significant financial losses (15). The presence of genotype VII strains in this region has emphasized the necessity for the development of a vaccine that is both efficacious and specifically tailored to the local conditions (16). Prior research has demonstrated that vaccines formulated utilizing local isolates can offer superior immunity in comparison to those derived from unrelated strains. Hence, this piece of work has aimed to develop and evaluate the immunogenicity of an inactivated ND vaccine produced from local isolates of genotype VII.2 in Bangladesh.

## 2 Material and methods

### 2.1 Strain selection, propagation and virus titration

The NDV strain (GenBank: OR924274.1) of genotype VII.2 was selected as a vaccine candidate after a comprehensive assessment of its molecular, serological, and biological characterization and pathogenicity (17). To achieve large-scale manufacturing, the selected NDV strain was propagated in 9-day-old seronegative embryonated chicken eggs (ECEs) via the allantoic cavity route. Centrifugation of the allantoic fluid (AF) at 2795×g for 10 minutes at 4°C was performed to remove non-viral embryonic debris from the collected AF, which resulted in a transparent fluid. The tenth passage of the NDV isolate was chosen for large-scale propagation. The collected AF was cultured in bacteriological, fungal, and mycoplasma growth media and examined daily for a week to detect any microbial growth. The purity of the NDV was confirmed using the RT-PCR test (18). The Reed and Muench technique (19) were employed to determine the 50% embryo infectious doses (EID_50_).

### 2.2 Virus inactivation and vaccine preparation

The AF containing NDV was inactivated by treating with 0.1% (v/v) formalin (37% formaldehyde stock solution) at a temperature of 30°C for 48 hours. Random samples from each batch were then injected into eggs and subjected to this process at least three times to confirm that the virus was indeed inactivated completely. Once fully inactivated, the viruses were mixed into an aqueous phase of Montanide (ISA 78 VG mineral emulsion, SEPPIC, France) at a ratio of 30:70 (w/w), following the manufacturer’s instructions. Each dose (0.5 ml) of the emulsified vaccine contained (10^8.67^ EID_50_) of the virus equivalent to 100 µg of protein per dose.

### 2.3 Sterility and safety tests of inactivated ND vaccine

The experimentally developed ND vaccine was overlaid on aerobic and anaerobic bacterial, fungal, and mycoplasma media. The culture plates were checked daily from day 1 to day 7^th^ for microbial development. Double the recommended doses of the experimentally developed inactivated ND vaccine were injected into the neck region through the subcutaneous (SC) route of ten 21-day-old seronegative chickens and five chickens as control. The vaccinated and control chickens were examined for 14 days after injection to detect aberrant vaccination-related reactions (18).

### 2.4 Efficacy determination of ND vaccine depending on dose and route of administration

In this study, we conducted an experiment using 140 of seven-day-old seronegative Sonali chicks to investigate the immune response of a newly developed inactivated ND vaccine. The main focus of this experiment was to determine the optimal dose and route of immunization. Chickens were divided into seven groups: six test groups (Group A, Group B, Group C, Group D, Group E, and Group F), and one control group (Group G) each group consisting of 20 birds. For the test groups, birds of groups A, B, and C received primary vaccination with 0.25 ml, 0.5 ml, and 1.00 ml per bird through the SC route, respectively. On the contrary, the birds of test groups D, E, and F also received 0.25 ml, 0.5 ml, and 1.00 ml per bird as primary vaccination in the thigh muscle through the intramuscular (IM) route, respectively. The control group (Group G) remained un-vaccinated. The initial vaccination of birds was done at 7 days old, followed by a second vaccination at the age of 28 days, except for the control group. Blood samples of birds of all groups were collected at three different time points: before vaccination (day 7), day 21^st^ after primary vaccination (day 28), and one month after second vaccination (Day 58). The serum obtained from blood samples of each bird group was pooled and subsequently analyzed for the determination of antibody titer by ELISA (IDEXX, USA) following the manufacturer’s instructions.

### 2.5 Evaluation of comparative efficacy and protective potentiality of the inactivated ND and commercial live ND vaccines

In this study, 200 seronegative layer chicks (Brown-layer) of 7 days old were used. Birds were divided into four groups, each group consisting of 50 birds. The vaccination protocol followed for each group of birds was as: Group A received two doses with an experimentally developed inactivated ND vaccine, each dose was 0.5 ml and contained 100 µg of viral protein per dose, administered through SC route. Group B was first vaccinated with a live vaccine (LaSota strain, genotype II), one drop containing 10^3.5^ EID_50_ of NDV, administered intraocularly (IO), followed by an inactivated ND vaccine with a similar dose as Group A. Group C was vaccinated twice with the commercial live vaccine (ND-LaSota/G-II, HIPRAVIAR^®^ S) each time receiving one drop of the same dosage and route followed for live vaccine as of Group B. Group D served as the unvaccinated control group. Birds of all the experimental groups received their first doses at 7 days of age (Day 7), and the second dose at 28 days of age (Day 28), except for the control group. Blood samples were collected from the birds prior to the primary and second vaccinations, as well as one month after the second vaccination. The serum samples were then tested to determine their antibody titer by ELISA. One month after second vaccination (Day 58) thirty birds of each group were subjected to a challenge with a virulent strain of NDV (Genotype VII.2). For the challenge study each bird of in the study groups received 0.5 ml containing 10^6.67^ EID_50_/ml of NDV, which was injected into the thigh muscle through the IM route. The birds were monitored daily for 14 days after the challenge to assess the protective efficacy of the experimentally developed inactivated ND vaccine and the live ND vaccine as well.

### 2.6 Determination of duration of antibody titer of the birds vaccinated with inactivated ND vaccine and commercial live ND vaccines

The remaining birds of the three previously vaccinated groups were assessed for the determination of serum antibody titers up to eight months after receiving second vaccinations. Blood samples were obtained at different time points: 1, 2, 3, 5, 6, 7, and 8 months after the second vaccination, and the titer was determined by ELISA.

### 2.7 Statistical analysis

The study was performed using computerized statistical software, especially SPSS (IBM SPSS Statistics for Windows, Version 29.0.2.0, Armonk, NY: IBM Corp, 2023). The data obtained from the three replications of different experimental groups in this study were presented as means and standard deviations (mean ± SD). A t-test was used to analyze the antibody titer between before vaccination versus after 21 days of primary vaccination, before vaccination versus after one month of second vaccination, and before vaccination versus different time intervals after primary and second vaccination in this study. The protection rate of the vaccine after the challenge study was also analyzed by one-way analysis of variance (ANOVA). The group difference was considered significant at *p < 0.05* in this study.

## 3 Results

### 3.1 Sterility and purity test of harvested AF

The sterility of the collected AF was performed using several types of agar media, including nutrient agar, SS agar, blood agar, EMB agar, MacConkey agar, Sabouraud dextrose agar, and mycoplasma agar (PPLO) test result revealed that there was absence of bacteria, fungi, or mycoplasma in the aseptically harvested AF. Additionally, PCR analysis using fusion (F) gene-specific primers confirmed the purity of the AF, which contained NDV only.

### 3.2 Inactivation, sterility, and safety tests of the experimentally developed ND vaccine

After being inoculated into 9-day-old ECEs, the absence of embryo mortality and hemagglutinating activity with cRBC of the inactivated AF indicated that the viruses had been inactivated completely. The sterility test also confirmed that the experimentally developed inactivated ND vaccine was free from any kind of contamination, as demonstrated by allowing the AF to overlay onto the media for bacteria, fungi, and mycoplasma. During the safety test performed in birds vaccinated with double the recommended doses of the experimentally developed ND vaccine and also in control birds, no deaths, local or systemic tissue reactions, or clinical signs of NDV were noted during 14 days of observation.

### 3.3 Dose and route-dependent antibody titer of the inactivated and live ND vaccines

**Figure 1:**
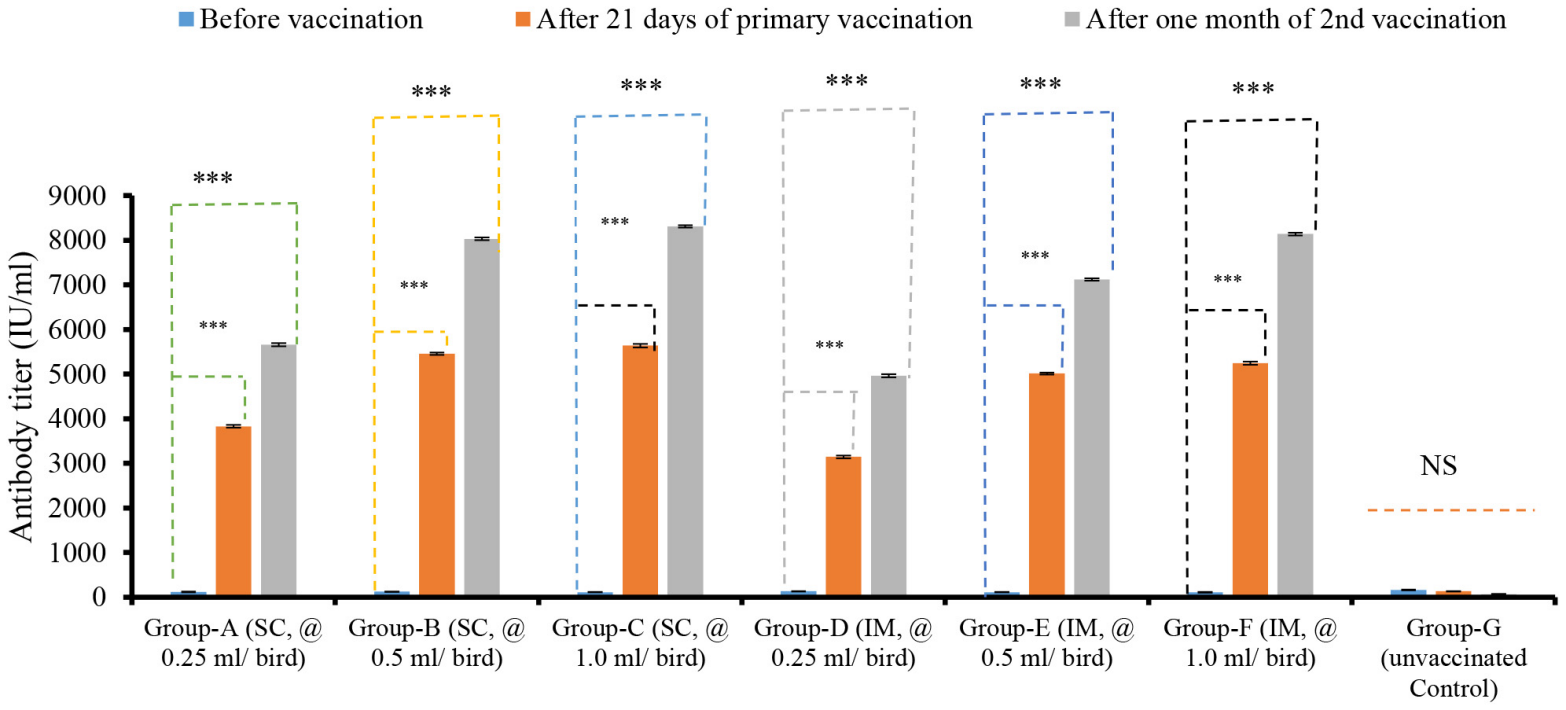
Dose and route-dependent antibody titers of bird vaccinated with ND vaccines by ELISA. NS indicates non-significant (*p>0.05*); *** indicates *p<0.001*.

The antibody titers varied across seven groups, depending on the doses and routes of vaccination. The antibody titers in birds of all groups before they were vaccinated ranged from 112.34 ± 3.51 IU/ml to 162.21 ± 4.05 IU/ml. The ELISA results showed that the serum antibody titers of the bird groups that were vaccinated after 21 days of primary and one month of second vaccinations through the SC route with 1.0 ml/bird group showed titers of 5638.02 ± 42.34 IU/ml and 8312.05 ± 24.26 IU/ml, with 0.5 ml/dose/bird at 5456.76 ± 25.70 IU/ml and 8033.45 ± 30.87 IU/ml, and with 0.25 ml/dose/bird group 3828.34 ± 31.41 IU/ml and 5656.24 ± 35.30 IU/ml, respectively. On the other hand, the antibody titer of the bird groups those received 1.0 ml/bird, 0.5 ml/bird, and 0.25 ml/bird through the IM route of immunization were 5246.14 ± 32.68 IU/ml, 8141.13 ± 32.14 IU/ml, 5012.36 ± 24.66 IU/ml, 7123.86 ± 26.60 IU/ml, and 3144.65 ± 34.20 IU/ml, 4962.68 ± 35.83 IU/ml, respectively (**Figure 1**). The antibody titers of birds were found to be significantly different (*p < 0.001*) between the period before vaccination and 21 days after the primary vaccination, as well as between the period before vaccination and one month after the second vaccination. After 21 days of primary and one month of second vaccination, the birds vaccinated through the SC route showed slightly higher antibody titers than the birds of the IM route group. The antibody titers of bird groups after primary and secondary immunizations between the SC and IM routes of immunization did not show a significant (*p > 0.05*) difference depending on the route of vaccination (**Figure 1**).

### 3.4 Comparative efficacy and protective potentiality of the experimentally developed inactivated ND and commercial live ND LaSota vaccines

**Figure 2:**
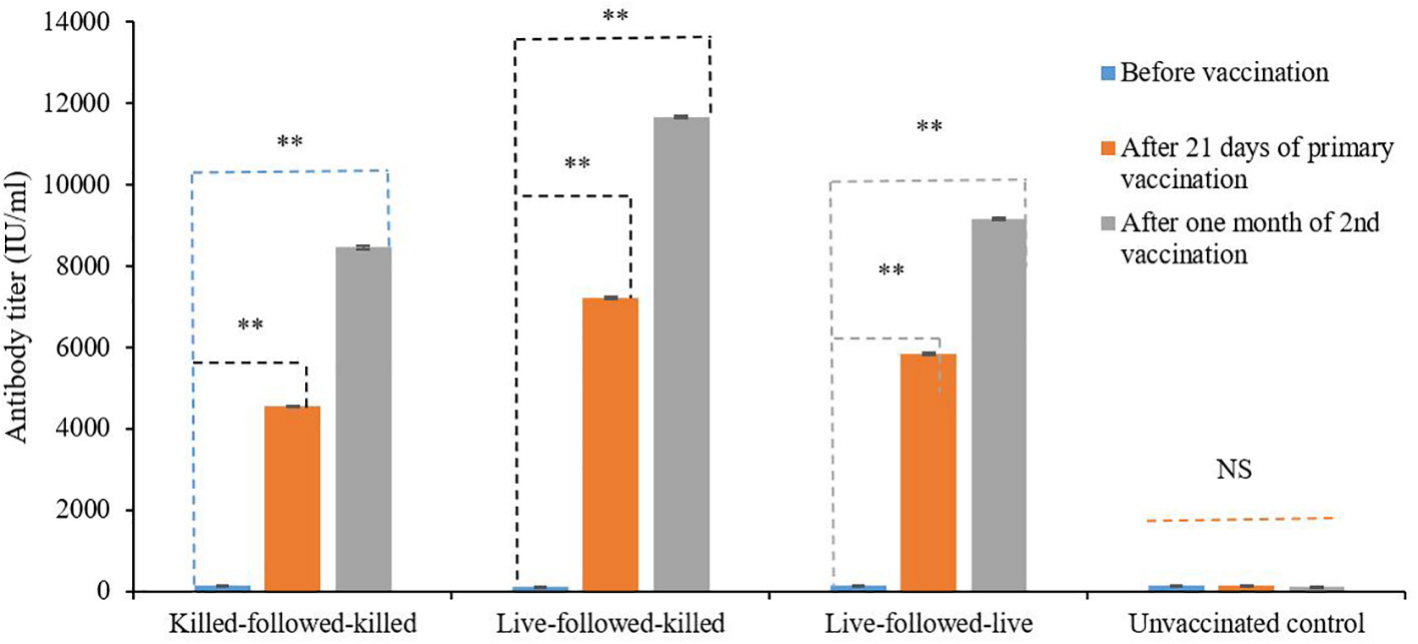
Serum antibody titer of birds vaccinated with the experimentally developed inactivated ND and commercial live ND LaSota vaccines by ELISA. NS indicates non-significant (*p>0.05*); ** indicates *p<0.01*.

The antibody titers of vaccinated groups of birds following three schedules of vaccination were determined by ELISA, which revealed that the pre-vaccination titers ranged from 132.7 ± 3.99 IU/ml to 144.1 ± 4.089 IU/ml. Whereas, the antibody titer was found to be higher 7235.87 ± 29.56 IU/ml and 11678.3 ± 30.17 IU/ml after 21 days of the primary and one month after second vaccinations in the birds group vaccinated with live-followed-killed vaccines (**Figure 2**). On the other hand, the antibody titers of the bird groups killed-followed-killed and live-followed-live were found relatively lower than live-followed-killed after 21 days of primary and one month after second vaccinations, which was statistically significant from prior vaccination (*p < 0.01*).

**Figure 3:**
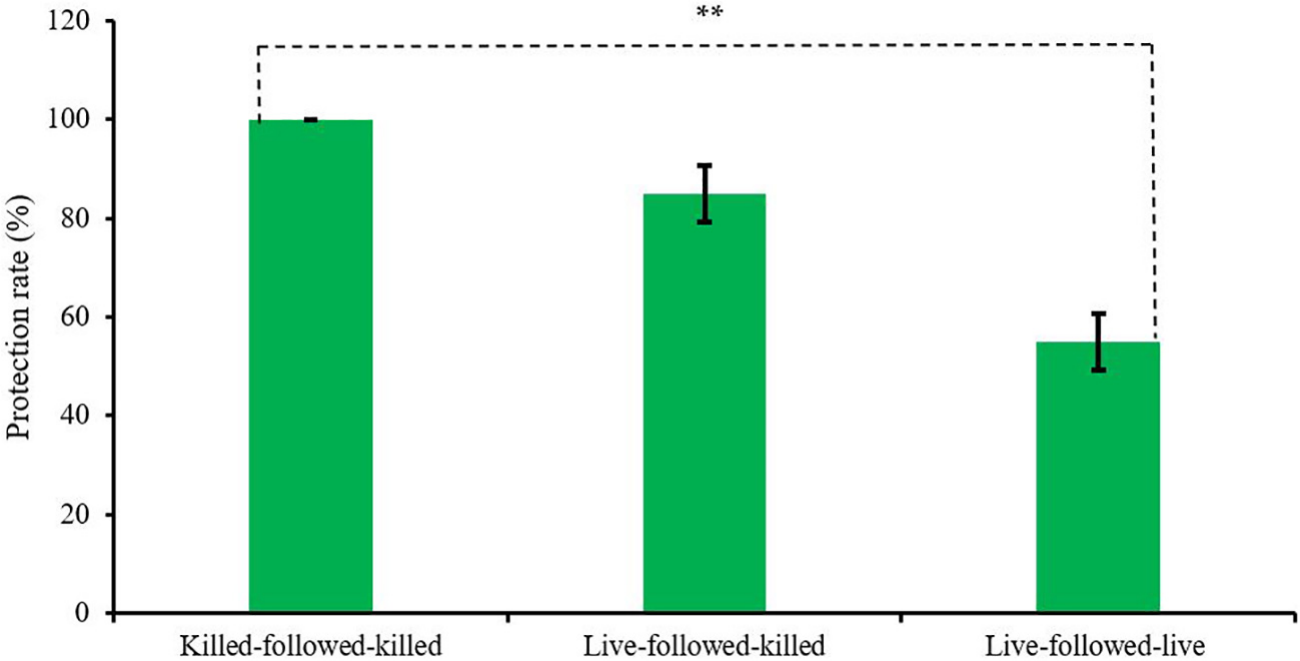
Protection rate of the experimentally developed inactivated ND and commercial live ND vaccines. (** indicates *p<0.01*).

The protection rates of birds among the three vaccinated groups, killed-followed-killed, live-followed-killed, and live-followed-live were also found variable 100 ± 0.00%, 83 ± 5.77%, and 57 ± 5.77%, respectively (**Figure 3**) which was statistically significant (*p < 0.01*).

### 3.5 Retention of the protective level of antibody titer of bird groups following three schedules of vaccination

**Figure 4:**
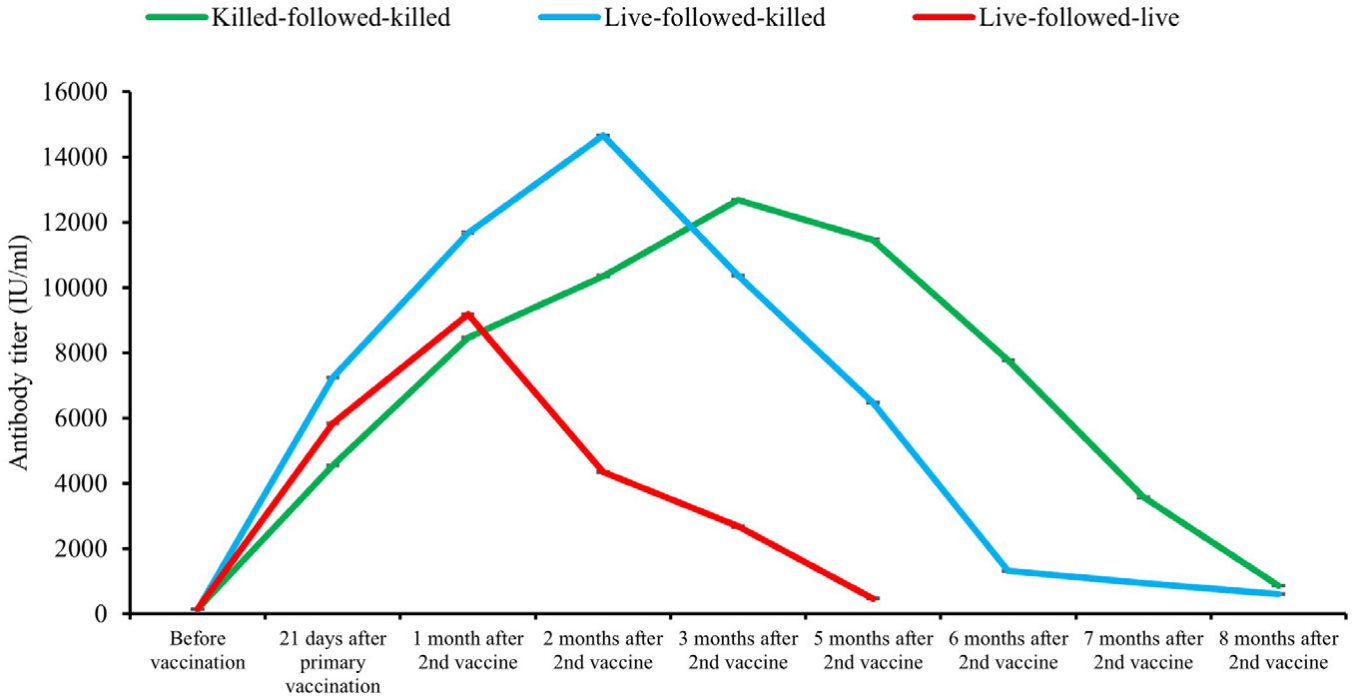
Duration of antibody titer of bird groups following three schedules of vaccination.

Pre-vaccination antibody titers of the birds of the groups killed-followed-killed, live-followed-killed, and live-followed-live groups were 144.1 ± 4.089 IU/ml, 132.7 ± 3.99 IU/ml, and 140.5 ± 3.83 IU/ml, respectively which was statistically non-significant (*p > 0.05*). The antibody titer of birds in three vaccination schedules showed significant variation (*p < 0.01*) after 21 days of the primary vaccination, and one and two months after second vaccination from before vaccination. The antibody titer of birds started declining gradually after three months of the second vaccination for the killed-followed-killed group, two months for the live-followed-killed group, and one month for the live-followed-live group. The retention period of protective level (>396 IU/ml) of serum antibody titer among the birds of three vaccinated groups, namely killed-followed-killed was until the 8^th^ month (856.2 ± 31.79 IU/ml), live-followed-killed until the 8^th^ month (609.5 ± 27.30 IU/ml), and live-followed-live until 5^th^ months (463.12 ± 29.69 IU/ml) after second vaccination, respectively (**Figure 4**). The declining trend of the antibody titer was found faster in the bird groups following the schedules live-followed-live and live-followed-killed compared to the birds of the group killed-followed-killed respectively. A significant variation (*p < 0.01*) in antibody titers was observed among the bird groups vaccinated with killed-followed-killed, live-followed-killed, and live-followed-live vaccines. This variation was evident on 21 days after the primary vaccination and one and two months after the second vaccination, compared to pre-vaccination antibody titer. However, the study also found no significant variation (*p > 0.05*) in the antibody titers at three, five, six, seven, and eight months after the second vaccination among the three different vaccination schedules.

## 4 Discussion

The genotype VII of the NDV leads to significant financial losses in the poultry sector due to increase death rates, decreased production, and augmented expenses for vaccination and biosecurity measures. The financial consequences are aggravated by trade restrictions and reduced consumer confidence, which notably affect small-scale farmers and worldwide market dynamics (20).

This investigation found that the ND vaccine made from recent circulating isolates was devoid of microbial contamination and other hemagglutinating and non-hemagglutinating viruses which were confirmed through sterility and purity tests by inoculating into various bacteriological, mycoplasmal, and fungal media and seronegative ECEs. The vaccine was prepared by mixing inactivated NDV antigen with Montanide ISA 78 VG adjuvant in a ratio of 30:70, consistent with the findings of Fawzy et al. (21). Their study showed that chickens immunized with the inactivated ND vaccine containing Montanide ISA 70 adjuvant. On the other hand, the vaccine without an adjuvant failed to provide any protection. The safety test demonstrated that birds injected with double the recommended doses of the experimentally developed ND vaccine did not experience any deaths, local or systemic tissue reactions, or clinical symptoms of ND. This result is in line with the findings of Aljumaili et al. (22) and Fawzy et al. (21), who examined the sterility, purity, and safety of the inactivated ND vaccine prepared with the genotype VII.2 in the same host system.

The present study evaluated the immune response of birds vaccinated with three different doses (0.25 ml/bird, 0.5 ml/bird, and 1.0 ml/bird) and two routes (SC and IM) of administration for the inactivated ND vaccine. After 21 days of primary and one month after second immunization, the serum antibody titers of birds vaccinated through the SC route with three different doses (1.0 ml/dose per bird, 0.5 ml/dose per bird, and 0.25 ml/dose per bird) showed slightly higher titers than the birds that were vaccinated by the IM route with similar doses. These findings are consistent with the findings of Woziri et al. (23), who developed an inactivated H5 vaccine and determined the antibody response in birds vaccinated with three different doses (0.2, 0.5, and 0.7 ml/bird) by two routes (IM and SC) of administration. In their study, they reported that the antibody levels were higher in the bird groups vaccinated through the SC route than the IM route after 21 days of post-vaccination. After initial screening for mass immunization, the dose and route (0.5 ml/dose/bird/SC) of inactivated ND vaccine developed with local isolates of NDV used to check their immune response in the vaccinated groups of birds (by ELISA) were selected as a standard dose because of its viral antigen concentration (10^8.67^ EID_50_/dose) and cost-effectiveness per dose.

In this study, birds immunized twice with commercial live-followed-killed ND vaccines showed higher serum antibody titers after three months of second vaccination than birds vaccinated with experimentally developed killed-followed-killed and commercial lived-followed-live. Live followed-by-killed vaccination in poultry is considered to be superior compared to the combination of killed followed by killed and live followed by live vaccination for several reasons. Live vaccines stimulate both cell-mediated and humoral immunity, providing robust and long-lasting protection (24). Administering a live vaccine first effectively primes the immune system, preparing it for a stronger response. Subsequent administration of a killed vaccine boosts this response without the risk of live pathogen replication. In contrast, killed vaccines alone primarily induce humoral immunity and may require multiple doses for adequate protection (25). Live-followed by live vaccination can pose higher risks, such as vaccine-associated disease or reversion to a virulent form of the agent. Therefore, the live-killed regimen strikes a balance, enhancing immunity while minimizing risks, and ensuring optimal health and productivity in poultry. The study agreed with the findings of Chansiripornchai and Sasipreeyajan (26) who reported that the antibody titer was found higher in the bird groups vaccinated with live-followed-killed vaccines than that of live followed by live ND vaccines. Their research also demonstrated that the optimal level of protection is attained by vaccinating birds in combination with live followed by killed vaccinations.

The birds that were vaccinated twice with the killed-followed-killed vaccine showed a higher protection rate (100%) compared to the birds of the groups live-followed-killed (83%) and live-followed-live (57%) groups respectively in this study. The efficacy study of Waheed et al. (27) revealed that broiler birds vaccinated with an inactivated vaccine developed with the locally isolated VG/GA strain of NDV showed 75% protection after being challenged at six weeks post-vaccination. Vaccines that have been developed by inactivation are known to elicit strong humoral and cell-mediated immune responses, providing efficient defense. Killed vaccines often exhibit better protective efficacy than live vaccines when challenged with a homologous organism due to their ability to consistently induce a strong and specific immune response (28). Killed vaccines contain inactivated pathogens that ensure a robust and safe antigen presentation without the risk of reverting to a virulent form also results inducing higher antibody titers and effective memory cell formation. Additionally, killed vaccines can be formulated to include adjuvants that enhance the immune response for a longer period. Their predictable nature and lack of interference from pre-existing immunity make killed vaccines a reliable option for inducing protective immunity against the same organism.

The protective level (>396 IU/ml) of serum antibody titer of bird groups of this study vaccinated twice with the experimentally developed inactivated ND vaccine was found to be retained up to eight months after second vaccination. The declining rate of antibody titer was found to be faster in the bird groups vaccinated with live-followed-live and live-followed-killed compared to the bird group killed-followed-killed in this study. The study supports the findings of Kumar et al. (29), who stated that inactivated vaccinations with genotype VII of NDV typically result in a long-lasting immune response, which is crucial for effectively managing ND in poultry populations over a longer period. Fawzy et al. (21) found that chicks vaccinated with inactivated ND vaccine developed with genotype VII of NDV had a serum antibody titer that peaked at the 6^th^-week post-vaccination, reaching the highest level (8.90 log^2^) after three weeks post-vaccination. The antibody titer remained at a protective level until the 16^th^ week post-vaccination and then declined gradually. The birds vaccinated with the inactivated ND vaccine showed their antibody titer peak at four weeks and remained at the protective level for up to 49 weeks post-vaccination (30, 31). These findings also align with Miller et al. (32), who found that genotype-matched vaccines developed with the field isolates can offer superior protection against ND. Their study also demonstrated that birds vaccinated with inactivated vaccines made from a locally circulating genotype developed homologous humoral immunity, which can reduce the amount of virus shed and increase protection rates compared to those of live ND vaccines. Sultan et al. (16) discovered that vaccinating commercial layers in combination with a recombinant genotype-matched inactivated vaccine and a live attenuated vaccine effectively reduced virus shedding and improved egg production when dealing with a genotype VII NDV in practical conditions. The study found that two doses of inactivated vaccine developed experimentally with the locally circulating genotype VII.2 of NDV elicited better immune response and provided better protection in the vaccinated flock of birds.

## 5 Conclusion

The results of the experiments suggest that the inactivated ND vaccine, which was developed with the locally circulating isolate of NDV, is not only safe but also protects birds from challenges with virulent field viruses. In addition to that, it also demonstrates the ability of the experimentally developed inactivated ND vaccine for successful management of the commercial layer poultry population from frequent of ND in Bangladesh. Development of an inactivated ND-vaccine using locally circulating isolate of the genotype VII.2 of NDV is the first time reported in Bangladesh.

## Data Availability

The datasets presented in this study can be found in online repositories. The names of the repository/repositories and accession number(s) can be found in the article/**Supplementary Material**.

## References

[1] Rehan M Aslam A Khan MR Abid M Hussain S Amber J. Potential economic impact of Newcastle disease virus isolated from wild birds on commercial poultry industry of Pakistan: A review. Hosts viruses. (2019) 6:1–5. doi: 10.17582/journal.hv/2019/6.1.1.15

[2] Petrone-García VM Castellanos-Huerta I Tellez-Isaias G. High-impact respiratory RNA virus diseases. Front Vet Sci. (2023) 10:1273650. doi: 10.3389/fvets.2023.1273650 37675076 PMC10478262

[3] Suarez DL Miller PJ Koch G Mundt E Rautenschlein S. Newcastle disease, other avian paramyxoviruses, and avian metapneumovirus infections. Dis Poult. (2020) 13:109–66. doi: 10.1002/9781119371199.ch3

[4] Hu S Ma H Wu Y Liu W Wang X Liu Y. A vaccine candidate of attenuated genotype VII Newcastle disease virus generated by reverse genetics. Vaccine. (2009) 27:904–10. doi: 10.1016/j.vaccine.2008.11.091 19095026

[5] Rahman M Chowdhury EH Parvin R. Small-scale poultry production in Bangladesh: challenges and impact of COVID-19 on sustainability. Ger J Vet Res. (2021) 1:19–27. doi: 10.51585/gjvr.2021.0004

[6] Ike AC Ononugbo CM Obi OJ Onu CJ Olovo CV Muo SO. Towards improved use of vaccination in the control of infectious bronchitis and Newcastle disease in poultry: understanding the immunological mechanisms. Vaccines. (2021) 9:20. doi: 10.3390/vaccines9010020 33406695 PMC7823560

[7] Dimitrov KM Afonso CL Yu Q Miller PJ. Newcastle disease vaccines-A solved problem or a continuous challenge? Vet Microbiol. (2017) 206:126–36. doi: 10.1016/j.vetmic.2016.12.019 PMC7131810 28024856

[8] Andey T Soni S Modi S. Conventional vaccination methods: Inactivated and live attenuated vaccines. Adv Vaccination Technol Infect Chronic Dis. (2024) 1:37–50. doi: 10.1016/B978-0-443-18564-9.00030-8

[9] Sarker MT Rahman MM Fakhruzzaman M Islam MN. Comparative efficacy of LaSota, B1 and mukteswar strain vaccines for Newcastle disease virus (NDV) in layer chickens. Asian J Med Biol Res. (2021) 7:332–8. doi: 10.3329/ajmbr.v7i4.57613

[10] Guérin JL Balloy D Pinson M Jbenyeni A Delpont M. Vaccination technology in poultry: principles of vaccine administration. Avian Dis. (2024) 67:489–94. doi: 10.1637/aviandiseases-D-23-99997 38300668

[11] Sedeik ME Elbestawy AR El-Shall NA Abd El-Hack ME Saadeldin IM Swelum AA. Comparative efficacy of commercial inactivated Newcastle disease virus vaccines against Newcastle disease virus genotype VII in broiler chickens. Poult Sci. (2019) 98:2000–7. doi: 10.3382/ps/pey559 30561723

[12] Duan Z Xu H Ji X Zhao J. Recombinant Newcastle disease virus-vectored vaccines against human and animal infectious diseases. Future Microbiol. (2015) 10:1307–23. doi: 10.2217/FMB.15.59 26234909

[13] Hu Z He X Deng J Hu J Liu X. Current situation and future direction of Newcastle disease vaccines. Vet Res. (2022) 53:99. doi: 10.1186/s13567-022-01118-w 36435802 PMC9701384

[14] Ellakany H El-Hamid A Nasef S Abdel Aziz M Gado A Zedan R. Evaluation of the protection of commercial live and inactivated NDV vaccines against Newcastle virus genotype VIId circulating in the field. Damanhour J Vet Sci. (2019) 1:17–20. doi: 10.5455/DJVS.25271

[15] Khatun M Islam I Ershaduzzaman M Islam HM Yasmin S Hossen A. Economic impact of Newcastle disease on village chickens-a case of Bangladesh. Asian Inst Res J Econ Bus. (2018) 1:358–67. doi: 10.31014/aior.1992.01.03.33

[16] Sultan HA Talaat S Elfeil WK Selim K Kutkat MA Amer SA. Protective efficacy of the Newcastle disease virus genotype VII–matched vaccine in commercial layers. Poult Sci. (2020) 99:1275–86. doi: 10.1016/j.psj.2019.10.063 PMC7587656 32111305

[17] Haque MA Sadekuzzaman M Haque ME Parvin MK Kamal MM Hayat S. Characterization of the dominant strain (G-VII) of Newcastle disease viruses isolated from commercial chickens in Bangladesh during recent outbreaks. J Adv Vet Anim Res. (2024) 11:407–17. doi: 10.5455/javar.2024.K790 PMC11296175 39101070

[18] WOAH. Manual of diagnostic tests and vaccines for Terrestrial animals (Mammals, Birds and Bees). 7th edition. Paris: World Health Organization (2021) p. 1–23.

[19] Reed LJ Muench H. A simple method of estimating fifty per cent endpoints. Am J Hyg. (1938) 27:493–5. doi: 10.1093/oxfordjournals.aje.a118408

[20] El-Dabae WH Ibrahim ES Sadek EG Kandil MM. preliminary evidence on passage attenuation of Orthoavulavirus javaense genotype VII. 1.1 and its potency as a living vaccine candidate. Adv Anim Vet Sci. (2024) 12:586–95. doi: 10.17582/journal.aavs/2024/12.4.586.595

[21] Fawzy M Ali RR Elfeil WK Saleh AA El-Tarabilli MM. Efficacy of inactivated velogenic Newcastle disease virus genotype VII vaccine in broiler chickens. In: Veterinary Research Forum, vol. 11. Faculty of Veterinary Medicine, Urmia University, Urmia, Iran (2020). p. 113. 32782739 10.30466/vrf.2019.95311.2295 PMC7413011

[22] Aljumaili OA Bello MB Yeap SK Omar AR Ideris A. Protective efficacy of inactivated Newcastle disease virus vaccines prepared in two different oil-based adjuvants. Onderstepoort J Vet Res. (2020) 87:1–7. https://hdl.handle.net/10520/ejc-opvet-v87-n1-a16. 10.4102/ojvr.v87i1.1865 PMC7565102 33054260

[23] Woziri AO Meseko CA Nasir FI Abdulkarim K Fasina FO Adamu J. Impact of dose and route of administration on antibody responses of chickens inoculated with inactivated Avian Influenza H5 vaccine. Microbes Infect Dis. (2022) 3:733–43. doi: 10.21608/mid.2021.72759.1149

[24] Vashishtha VM Kumar P. The durability of vaccine-induced protection: An overview. Expert Rev Vaccines. (2024) 23:389–408. doi: 10.1080/14760584.2024.2331065 38488132

[25] Amanna IJ Slifka MK. Contributions of humoral and cellular immunity to vaccine-induced protection in humans. Virology. (2011) 411:206–15. doi: 10.1016/j.virol.2010.12.016 PMC3238379 21216425

[26] Chansiripornchai N Sasipreeyajan J. Efficacy of live B1 or Ulster 2C Newcastle disease vaccines simultaneously vaccinated with inactivated oil adjuvant vaccine for protection of Newcastle disease virus in broiler chickens. Acta Vet Scand. (2006) 48:1–4. doi: 10.1186/1751-0147-48-2 PMC1513127 16987398

[27] Waheed U Siddique M Arshad M Ali M Saeed A. Preparation of newcastle disease vaccine from VG/GA strain and its evaluation in commercial broiler chicks. Pakistan J Zool. (2013) 45:399–44.

[28] Wilson-Welder JH Torres MP Kipper MJ Mallapragada SK Wannemuehler MJ Narasimhan B. Vaccine adjuvants: current challenges and future approaches. J Pharm Sci. (2009) 98:1278–316. doi: 10.1002/jps.21523 PMC8092333 18704954

[29] Kumar BSA Panickan S Bindu S Kumar V Ramakrishnan S Saxena S. Immunogenicity and protective efficacy of an inactivated Newcastle disease virus vaccine encapsulated in poly-(lactic-co-glycolic acid) nanoparticles. Poult Sci. (2023) 102:10–26. doi: 10.1016/j.psj.2023.102679 PMC10160591 37116285

[30] Andrews SJ Poole EJ Stuke K Salt J. Duration of immunity of live and inactivated Newcastle disease vaccines in SPF chickens following a single administration. Edinburgh, UK: Galvmed (2021) p. 1–10.

[31] Siddique F Abbas RZ Iqbal A Rabbani M Rafique A Hussain I. Development of humoral immune response to thermostable Newcastle disease vaccine strain i-2 in ring-necked pheasant (*Phasianus colchicus*). Kafkas Univ Vet Fak Derg. (2021) 27:253–8. doi: 10.9775/kvfd.2020.25021

[32] Miller PJ Afonso CL El Attrache J Dorsey KM Courtney SC Guo Z. Effects of Newcastle disease virus vaccine antibodies on the shedding and transmission of challenge viruses. Dev Comp Immunol. (2013) 41:505–13. doi: 10.1016/j.dci.2013.06.007 23796788

