# Supplementary material for: Determination of immunogenicity of an inactivated ND-vaccine developed experimentally with Newcastle disease virus (Genotype VII.2) local isolates of Bangladesh
Source: Front Immunol. 2024 Nov 6;15:1482314. doi: 10.3389/fimmu.2024.1482314 (PMC11576377; doi:10.3389/fimmu.2024.1482314)
Supplement: Supplementary file 1 [file DataSheet1.docx]

Supplementary data for **Figure 1** Dose and route-dependent antibody titers of bird vaccinated with ND vaccines by ELISA.

| **Groups** | **Before vaccination** | **After 21 days of primary vaccination** | **After one month of 2^nd^ vaccination** |
| --- | --- | --- | --- |
| Group-A (SC, @0.25 ml/ bird) | 116 ± 7.09 | 3828.34 ± 31.41 | 5656.24 ± 35.30 |
| Group-B (SC, @ 0.5 ml/ bird) | 124± 6.08 | 5456.76 ± 25.70 | 8033.45 ± 30.87 |
| Group-C (SC, @ 1.0 ml/ bird) | 111± 4.06 | 5638.02 ± 42.34 | 8312.05 ± 24.26 |
| Group-D (IM, @0.25 ml/ bird) | 134± 6.11 | 3144.65 ± 34.20 | 4962.68 ± 35.83 |
| Group-E (IM, @0.5 ml/ bird) | 112± 5.21 | 5012.36 ± 24.66 | 7123.86 ± 26.60 |
| Group-F (IM, @1.0 ml/ bird) | 110± 6.14 | 5246.14 ± 32.68 | 8141.13 ± 32.14 |
| Group-G (unvaccinated Control) | 162± 5.43 | 132± 6.34 | 67± 5.75 |

Supplementary data for **Figure 2** Serum antibody titer of birds vaccinated with the experimentally developed inactivated ND and commercial live ND LaSota vaccines by ELISA.

| **Group** | **Before vaccination** | **After 21 days of primary vaccination** | **After one month of 2^nd^ vaccination** |
| --- | --- | --- | --- |
| Killed-followed-killed | 144.1±4.089 | 4545.34 ±27.64 | 8463.94 ±36.84 |
| Live-followed-killed | 132.7±3.99 | 7235.87±29.56 | 11678.3±30.17 |
| Live-followed-live | 140.5±3.83 | 5834.68±31.79 | 9174.83±30.13 |
| Unvaccinated control | 138±3.44 | 153±3.85 | 122±5.48 |

Supplementary data for **Figure 3** Protection rate of the experimentally developed inactivated ND and commercial live ND vaccines.

| **Groups** | **Protection rate (%)** |
| --- | --- |
| Killed-followed-killed | 100±0.00 |
| Live-followed-killed | 83.33±5.77 |
| Live-followed-live | 56.67±5.77 |

Supplementary data for **Figure 4** Duration of antibody titer of bird groups following three schedules of vaccination

| Sample | Before vaccination | 21 days  after 1^st^ vaccine | 1 month after 2^nd^ vaccine | 2 months after 2^nd^ vaccine | 3 months after 2^nd^ vaccine | 5 months after 2^nd^ vaccine | 6 months after 2^nd^ vaccine | 7 months after 2^nd^ vaccine | 8 months after 2^nd^ vaccine |
| --- | --- | --- | --- | --- | --- | --- | --- | --- | --- |
| Killed-followed  killed | 144.1  ±  4.089 | 4545.34 ±  27.64 | 8463.94 ±  36.84 | 10345.92  ± 40.56 | 12675.47  ±  30.99 | 11456.69  ± 35.30 | 7768.43  ± 32.72 | 3564.73  ± 35.42 | 856.2  ± 31.79 |
| Live-followed  killed | 132.7  ±  3.99 | 7235.87  ±  29.56 | 11678.3  ±  30.17 | 14658.84  ±  35.00 | 10366.76  ±  32.17 | 6458.67  ±  34.82 | 1314.56  ±  32.98 | 946.8±24.80 | 609.5±27.30 |
| Live-followed  live | 140.5  ±  3.83 | 5834.68  ±  31.79 | 9174.83  ±  30.13 | 4346.76  ±  32.94 | 2672.52  ±  34.73 | 463.12  ±  29.69 | - | - | - |
